# Portraits of breast cancer progression

**DOI:** 10.1186/1471-2105-8-291

**Published:** 2007-08-06

**Authors:** Gul S Dalgin, Gabriela Alexe, Daniel Scanfeld, Pablo Tamayo, Jill P Mesirov, Shridar Ganesan, Charles DeLisi, Gyan Bhanot

**Affiliations:** 1 Mol. Bio., Cell. Bio. and Biochem. Prog., Boston University, Boston, MA 02215, USA; 2 The Broad Institute of MIT and Harvard, 7 Cambridge Center, Cambridge MA, 02142, USA; 3 The Simons Center for Systems Biology, Institute for Advanced Study, Princeton, NJ 08540, USA; 4 Cancer Institute of New Jersey, 195 Little Albany Street, New Brunswick, NJ 08903, USA; 5 Center for Advanced Genomic Technology, Department of Biomedical Engineering, Boston University, Boston, MA 02215, USA; 6 BioMaPS Institute and Department of Biomedical Engineering, Rutgers University, Piscataway, NJ 08854, USA

## Abstract

**Background:**

Clustering analysis of microarray data is often criticized for giving ambiguous results because of sensitivity to data perturbation or clustering techniques used. In this paper, we describe a new method based on principal component analysis and ensemble consensus clustering that avoids these problems.

**Results:**

We illustrate the method on a public microarray dataset from 36 breast cancer patients of whom 31 were diagnosed with at least two of three pathological stages of disease (atypical ductal hyperplasia (ADH), ductal carcinoma in situ (DCIS) and invasive ductal carcinoma (IDC). Our method identifies an optimum set of genes and divides the samples into stable clusters which correlate with clinical classification into Luminal, Basal-like and Her2+ subtypes. Our analysis reveals a hierarchical portrait of breast cancer progression and identifies genes and pathways for each stage, grade and subtype. An intriguing observation is that the disease phenotype is distinguishable in ADH and progresses along distinct pathways for each subtype. The genetic signature for disease heterogeneity across subtypes is greater than the heterogeneity of progression from DCIS to IDC within a subtype, suggesting that the disease subtypes have distinct progression pathways.

Our method identifies six disease subtype and one normal clusters. The first split separates the normal samples from the cancer samples. Next, the cancer cluster splits into low grade (pathological grades 1 and 2) and high grade (pathological grades 2 and 3) while the normal cluster is unchanged. Further, the low grade cluster splits into two subclusters and the high grade cluster into four. The final six disease clusters are mapped into one Luminal A, three Luminal B, one Basal-like and one Her2+.

**Conclusion:**

We confirm that the cancer phenotype can be identified in early stage because the genes altered in this stage progressively alter further as the disease progresses through DCIS into IDC. We identify six subtypes of disease which have distinct genetic signatures and remain separated in the clustering hierarchy. Our findings suggest that the heterogeneity of disease across subtypes is higher than the heterogeneity of the disease progression within a subtype, indicating that the subtypes are in fact distinct diseases.

## Background

One out of ten women who reaches the age of ninety will have had breast cancer in her lifetime. Most tumors are treated with a combination of surgery, radiation therapy, and adjuvant systemic therapy (hormonal therapy, chemotherapy, and/or biological therapy). 60–80% of tumors express the estrogen receptor ER and respond to treatment with hormonal agents such as aromatase inhibitors or Tamoxifen [1,2]. 20–40% have amplification of the Her2 gene [3] which is a marker of increased recurrence rates and poorer prognosis. The outcome of these Her2+ tumors can be improved by the addition of the humanized anti-Her2 antibody trastuzumab (Herceptin) to their treatment regimen. 10–15% of tumors neither express the estrogen receptor nor harbor Her2 amplification and have a characteristic gene expression profile [4]. These cancers, called Basal-like [5,6], are high grade aggressive malignancies with poor overall prognosis, and at present there is no targeted therapy for them. In spite of these classifications and treatment choices, therapy is confounded by the fact that tumors with similar histopathology often have divergent course and varied response to therapy [7].

Microarrays have the potential to shed light on this picture because of their ability to provide a snapshot of the genetic state of the cell. In principal, they should be able to identify the genes and pathways altered in cancer initiation, progression and metastasis. This promise has resulted in microarray technology being aggressively pursued by researchers, hospitals and pharmaceutical companies to get an improved understanding of the disease process, better diagnostic protocols, new drugs, and new treatment regimens. However, the success of these efforts has been limited by practical considerations. The biggest limitation is that the results from microarray studies are sensitive to noise and analysis method [8]. This often leads to ambiguous results and biologically non-intuitive genes and pathways for stratification [8]. Efforts to use microarray data to identify the underlying biology of disease progression and help characterize the disease phenotype have met with limited success. In this paper, we develop and give results from a robust method which addresses the issues outlined above. We first use Principal Component Analysis (PCA) [9] to identify the overall structure of clusters in the data and to select the subset of genes that distinguish the clusters. We then use this set of genes and a new consensus ensemble *k*-clustering technique, which averages over several clustering methods and many data perturbations, to identify strong, stable clusters. We also define a simple criterion to find the optimum number of clusters and a method to identify robust markers for disease progression within each cluster.

## Results

Applied to a breast cancer microarray data set, our method results in stable lists of genes and pathways that distinguish high and low grade tumors. It also identifies other robust gene sets which mark progression of disease from DCIS or *ductal carcinoma in-situ *to IDC or *invasive ductal carcinoma*. The clusters paint a portrait of the disease at varying levels of granularity. When the data is divided into two clusters, the normal samples form one cluster and the disease samples form another. At the next level of clustering, the low grade and high grade samples separate. The optimal number of clusters is seven, corresponding to two sub-clusters (LG1 and LG2) of the low grade samples and four (HG1-HG4) of the high grade samples. These sub-clusters are well separated by a strong set of markers which are able to distinguish them with sensitivity and specificity in the 80–100% range. We identify the genes and pathways that mark disease progression in each sub-cluster. A major result of our analysis is that *each sub-cluster contains samples from non-invasive and invasive tumors from the same patient*. This suggests that within each grade of breast cancer, different groups of patients progress to the same final phenotype along different pathways. This result suggests that the sub-clusters identified here are distinct diseases. If validated on larger datasets with larger gene-sets, it would have significant implications for disease identification and treatment.

Using the genes specific to each cluster and ER, PR, Her2+ levels in the data, we can place the clusters into the standard categories used to classify breast cancer as defined in [5]. We find that the low grade clusters correspond to one Luminal A subtype and one Luminal B subtype. The high grade samples correspond to two additional Luminal B subtypes, one Her2+ subtype and one Basal-like subtype.

### Description of data

The data was obtained from [10] and consisted of samples from 36 breast cancer patients of which 31 were diagnosed with at least two out of three pathological stages of disease: atypical ductal hyperplasia or ADH, ductal carcinoma *in situ *or DCIS and invasive ductal carcinoma or IDC respectively. The remaining 5 patients were diagnosed to have pre-invasive disease (ADH) only. Microarray analysis was also done for samples collected from normal breast epithelial tissue extracted from three healthy women during routine mammoplasty. From the cancer patients, normal as well as disease samples were collected from as many different stages of disease (ADH, DCIS, IDC) as possible. These samples were harvested in triplicate using laser capture micro-dissection (Arcturus, CA), taking care to avoid contamination between cells of different stages from the same patient. Each sample was analyzed in duplicate with a 12,000 gene cDNA microarray. It was determined that the "normal cells" from cancer patients were highly similar to the normal epithelium of the three disease free patients. This suggested that the normal samples from cancer patients could be used as a baseline to determine disease state and progression.

The data provided in [10] consisted of the expression levels of 1940 genes across 93 samples. 32 of these were from disease free or normal tissue, 8 were ADH samples, 30 were DCIS samples and 23 were IDC samples. The 1940 genes (out of the 12,000 genes on the microarray) were selected in [10] by their ability to distinguish "normal cells" and each of the disease stages ADH, DCIS and IDC using a linear discriminant function. The patients were further classified by pathological analysis into 3 categories based on the tumor grade: grade I (18 patients), grade II (22 patients) and grade III (19 patients). The mapping of sample labels to stage and grade and a patient identifier is given in Additional File 1. This table also includes the classification of the samples into the disease subtypes using the methods described in this paper.

**Figure 1:**
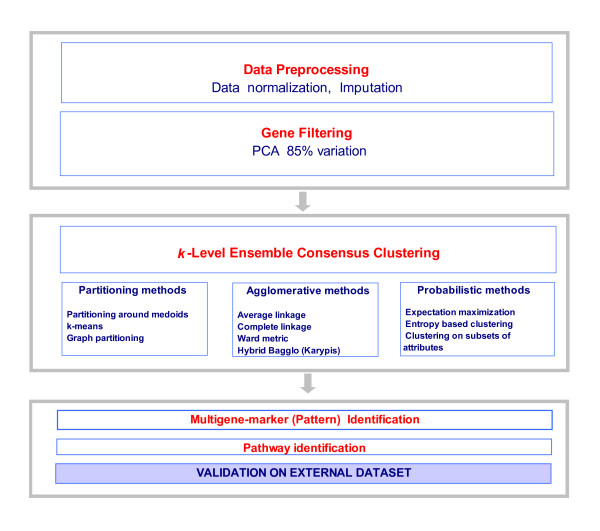
**Flow chart of the analysis method**: The method starts with data normalization and proceeds to the identification of predictive genes using principal component analysis to ensemble clustering into *k *= 2,3,... clusters. The clusters are then analyzed to identify their characteristic gene patterns which are then used to find altered pathways associated with the disease process.

The flow chart of our analysis method is presented in Figure 1. First the dataset was normalized and missing entries imputed robustly. Next, PCA was used to find the genes which accounted for most of the variation in the data. The optimal number of clusters *k*_opt _in the data was estimated using gap statistics [11] and silhouette scores [12]. Next a variety of clustering techniques and data perturbations are averaged to divide the data into 2,3,...*k*_opt _clusters.

We estimate the number of clusters using the silhouette scores and gap statistics. This provides a range for the number of clusters. In our case, the range was 6–7 so we chose *k*_max _= 7. This range is compared to the optimum number of clusters provided by the EM mixture modeling approach. (in Monti et al [17], AutoClass was used for this analysis. In our study we use the EMclust package in R [13]). We then create ensemble consensuses of *k *clusters for each *k *= 2, 3,..*k*_max _as follows:

For each *k*, we integrate the clustering results across clustering methods and perturbations into an agreement matrix. To obtain a global optimum clustering solution, we apply simulated annealing to the agreement matrix at each *k *to sort the samples into the best *k *clusters, which appear as blocks along the diagonal in the sorted agreement matrix. The agreement matrix cost function used in simulated annealing optimizes between the similarity inside the clusters and the dissimilarity between different clusters for each *k*. Once the data is clustered by simulated annealing into *k *pieces, we compute the silhouette scores for these *k *clusters (in addition to other measures such as the internal diameter, external dissimilarity etc) using the fpc package from R.

We stop at *k *= *k*_opt _clusters beyond which the "quality" of the clusters obtained from the agreement matrix begins to deteriorate. This is assessed by analyzing the accuracy of cluster membership assignments using weighted voting and kNN on single and multiple gene markers (patterns) which distinguish the clusters. We stop clustering when the accuracy of such an assignment falls below 75%.

### Principal Component Analysis

PCA showed that 50% of the variation in the data was represented by the first 5 PCs and 85% by the first 32 PCs. We identified 207 genes as those with highest absolute value (top 1st quartile) in the coefficients of the first 32 eigenvectors as representative of most of the data variation. These thresholds were estimated through a calibration step whose aim was to optimize the overall cluster membership assignment for the optimal number of clusters identified in the data restricted to the selected genes.

### Consensus ensemble *k*-clustering

Gap statistic and the silhouette scores [11,12] estimated *k *= 7 as the optimal value for the number of clusters in the data. The data was divided into *k *= 2, 3,...,7 clusters by using the 207 genes identified by PCA and by applying Consensus Ensemble *k*-clustering (see the **Methods **section below).

**Figure 2:**
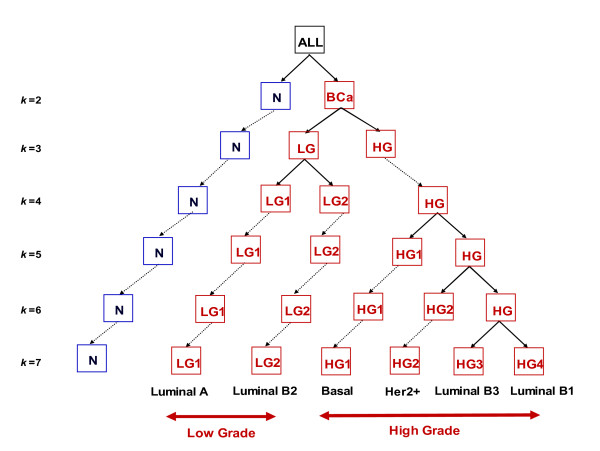
**Hierarchical nature of breast cancer progression**: Consensus ensemble *k*-clustering tree reveals the recursive splitting of breast cancer subtypes. At *k *= 2, the ensemble clustering split the normal samples from the disease samples. At *k *= 3, the normal cluster remained unchanged and the disease samples split into low grade (pathological grades 1 and 2) and high grade (pathological grades 2 and 3). The optimum number of clusters in the data was seven corresponding to one normal cluster, two low grade clusters and four high grade clusters. Between two *k *values, the samples did not switch clusters, indicating that the hierarchical structure in the figure is a strong property of the data. In the final disease clusters, samples from the same patient microdissected from DCIS and IDC lesions were found in the same cluster, indicating that the disease subtypes are more heterogeneous than disease progression within a subtype.

The results are shown schematically in Figure 2. At *k *= 2, the samples separated into a "normal" (N) group, which contained all the normal samples and one ADH sample (from patient id 210), and a "breast cancer" (BCA) group, which contained all the remaining breast cancer samples. At *k *= 3, the normal group was unaltered but the BCA group split into a low grade (LG) tumor group containing 18 samples labeled grade 1 and 9 samples labeled grade 2, and a high grade (HG) tumor group containing 13 samples labeled grade 2 and 19 samples labeled grade 3. As *k *increased progressively from 4 through 7, the LG group split into 2 distinct subgroups (labeled LG1 and LG2 in Figure 2) and the HG group split into 4 distinct subgroups (labeled HG1-HG4).

Even though the clusters at each level were determined independently, at clustering level *k*+1, two clusters always emerged as splits of a parent cluster at level *k*, while the remaining *k*-1 clusters were inherited unchanged from the previous level *k*. This shows that the data inherently contains a hierarchy of detail, providing portraits of disease at different levels of clustering resolution. The separation of samples into "normal" and "disease" at *k *= 2, the split of the disease samples into "low" and "high" grades at *k *= 3 and so on, strongly suggests that disease progression is a hierarchical process and is readily and robustly identifiable by our clustering procedure.

**Figure 3:**
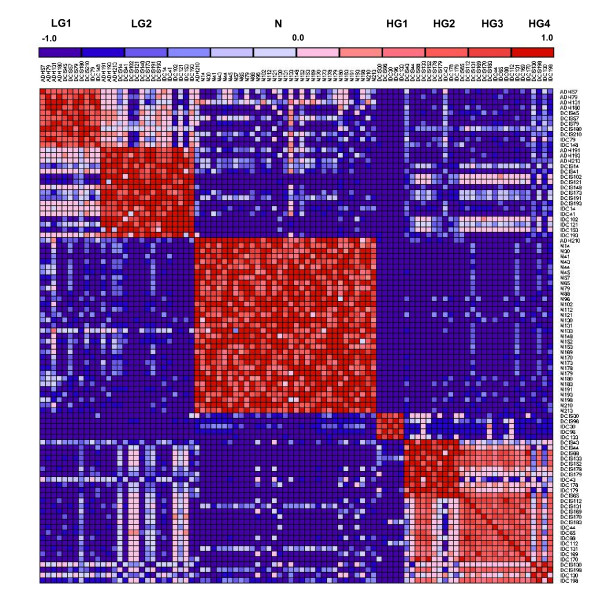
**Heatmap of agreement matrix for seven clusters**: The agreement matrix for N_S _samples is an N_S _× N_S _matrix whose entries are the fraction of cases across replicates for which two samples fall into the same cluster. Red/green represent high/low fractional values across clustering methods and data perturbation replicates. The normals and the LG1 and LG2 are clearly well separated while the HG1, HG2, HG3 and HG4 separation is weaker. We find that the optimum number of clusters using gap-statistics oscillates between 6 and 7 with the HG3 and HG4 clusters merging at *k*-6.

Table 1 shows the characteristics of the samples in these groups with respect to stage, ER, PR, Her2, lymph node and grade status for *k *= 2,3 and 7. These subgroups of LG and HG are strongly dissimilar with respect to the cluster agreement matrix, which is shown in Figure 3. The HG1 subgroup is particularly different from the other HG subgroups (as is also evident in Figure 2). All samples in it are ER-, PR- and mostly Her2-. The HG2 subgroup has a mixed ER signature, and the HG3 and HG4 subgroups consist mostly of ER positive samples. Based on these and other signatures (see below), we identify LG1 as Luminal A; LG2, HG3, HG4 as Luminal B; HG1 as Basal-like and HG2 as Her2+.

**Table 1:** Clinical characteristics of *k *= 2,3,7 clusters: The ER, PR, Her2, Node, stage and grade status of the samples in each cluster are shown for *k *= 2,3 and 7. ND stands for "Not Determined". At *k *= 2, the clustering splits the data into normal samples and disease samples (BCA), except for one ADH which is classified with the normals. At *k *= 3, the BCA samples split into high grade (grade 2 or 3) and low grade (grade 1 or 2) categories. At *k *= 7, the low grade samples split into two clusters LG1, LG2 and the high grade into four: HG1 – HG4. The HG1 samples are all ER-, PR- and mostly Her2-. The HG3 and HG4 clusters are mostly ER+, PR+, Her2-. The HG2 cluster has mixed ER, PR and Her2 signatures. Using the Sorlie et al classification, we identify HG1 as the Basal-like subtype; LG1 as Luminal A; LG2, HG3 and HG4 as Luminal B and HG2 as the Her2+ subtype. When the sum of the entries for ER/PR/Her2/Node/Grade do not add up to the size of the cluster, it is because the corresponding information was missing in the dataset [10].

Cluster level *k*	Group	Size	Stage				ER			PR			Her2			Node		Grade		
			
			ADH	DCIS	IDC	N	Pos	Neg	ND	Pos	Neg	ND	Pos	Neg	ND	Pos	Neg	1	2	3
2	N	33	1			32														
	BCA	60	7	30	23		47	10	3	42	15	3	10	37	9	44	14	18	22	19
3	LG	28	7	13	8		26		2	21	5	2	4	18	6	20	8	18	9	
	HG	32		17	15		21	10	1	21	10	1	6	19	3	24	6		13	19
7	LG1	11	4	5	2		11			8	3		1	10		7	4	9	2	
	LG2	17	3	8	6		15		2	13	2	2	3	8	6	13	4	9	7	
	HG1	5		2	3			5			5		1	4		3				5
	HG2	10		7	3		7	3		5	5		3	4	1	9	1		2	8
	HG3	13		6	7		10	2	1	12		1	2	7	2	10	3		7	6
	HG4	4		2	2		4			4				4		2	2		4	

### Genes discriminating low and high grade tumors

Using a non-stringent Signal-to-Noise-Ratio (SNR) test (permutation p-value p = 0.10) we found 223 gene markers which distinguish the group LG from HG. A subset of 10 markers was selected based on their performance on leave-one-out cross-validation experiments for weighted voting (WV) and *k*-Nearest Neighbors (kNN) classification models. The models trained on these 10 markers produced only 1 false positive error (DCIS #79) and 1 false negative error (DCIS #183) in leave-one-out experiments.

Using the SNR test and leave-one-out experiments for the WV and kNN models, we identified 10 markers which distinguish the LG samples from all others (HG and N) with 90% accuracy. We find that RBSK, Homo sapiens cDNA FLJ12924 fis, clone NT2RP2004709 and CRIP1 are up-regulated in the LG group, and EYA2, ANXA1, RUNX3, DKFZp762A227, GPRC5B are down-regulated in the LG group.

For the high grade cluster, the classification accuracy was 97% with 3 false positive and zero false negative errors. The top markers up-regulated in HG are TRAM, HSPC150, TACC3, CDKN3, UBE2C, and top markers down-regulated in HG are X123, GNG7, SH3BGRL2, LOH11CR2A and Homo sapiens, clone IMAGE:3917549 mRNA, partial cds.

### Low grade substructure

Table 1 shows that both LG1 and LG2 are ER+, PR+ and Her2-, which explains their pathological classification as low grade. We note that LG2 has a greater fraction of Grade II samples compared to LG1 which identifies LG2 as the more aggressive subtype. The genes that discriminate LG1 from other low and high grade subgroups include the down-regulated BIRC5 (survivin) gene, which inhibits apoptosis and is suggested as a marker of poor prognosis in different cancer types [14,15]. Two others are ACAA1 and ACOX1 enzymes, which are involved in fatty acid metabolism. LG2 markers include 190 genes, among which are many oncogenes, (BCL2 (down, breast cancer poor prognosis marker), RAD51 (up), EGFR (up), RUNX3 (up), BCL9 (down) and VAV3 (down) and tumor suppressor gene NME1 (up). The ER and Her2 status suggest that both LG1 and LG2 are Luminals in the standard nomenclature [5], with LG2 presenting more aggressive features than LG1. On the basis of this, in the nomenclature of [5], we identify LG1 as the Luminal A subtype and LG2 as one Luminal B subtype.

### High grade substructure

As seen in Table 1, all the samples in the HG1 subgroup were ER and PR negative while those in the HG3 and HG4 subgroups were mostly ER and PR positive. The HG2 samples had mixed ER and PR signatures. The HG1 subgroup, which is the worst prognosis group based on clinical characteristics, had as discriminatory markers the genes BCL2 (up), RAD51 (down), GSTP1 (down) and RRM2 (down). HG2 markers also include up-regulated BCL2 (1.7 fold less up-regulated than in HG1) and down-regulated RRM2.

The HG3 markers, include a group of down-regulated genes in chromosomal region 17q23-25 which harbors the ERBB2 amplicon 17q 22.24. These genes are KPNA2 (17q23.1-q23.3), amplified in breast cancer 1 (AIBC1, 17q23.2), Bcl-2 inhibitor of transcription (BIT1, 17q23.2), hypothetical protein TANC2 (17q23.3), and two proteosome protein PSMC5 (17q23-Q25) and PSMD12 (17q24.2). This suggests the possibility that patients in the HG3 subgroup might have a re-arrangement or deletion of genes around the Her2 gene leading to loss of regulation or function for these genes which might explain why only 15% of HG3 patients are Her2+, while 53% are Her2- and 15% are undetermined. Since IHC data for the ERBB2 gene was not made available in [10], the down-regulation of the ERBB2 amplicon genes in the HG3 samples identifies its clinical signature as Her2-.

The most notable HG4 marker was a down-regulation in the transforming growth factor beta receptor II (TGFBR2). Mutations in this gene have been associated with the development of various types of tumors. The over-expression of this gene was found to be associated with poor prognosis breast tumors. Overall, gene markers and clinical parameters lead to the conclusion that among the high grade subgroups HG4 is probably the best prognosis group composed of Grade II tumors that are all ER+ and PR+.

Based on these observations, we identify HG1 as Basal-like [5,6], HG2 as Her2+, and HG3 and HG4 as additional subtypes of Luminal B [5].

**Figure 4:**
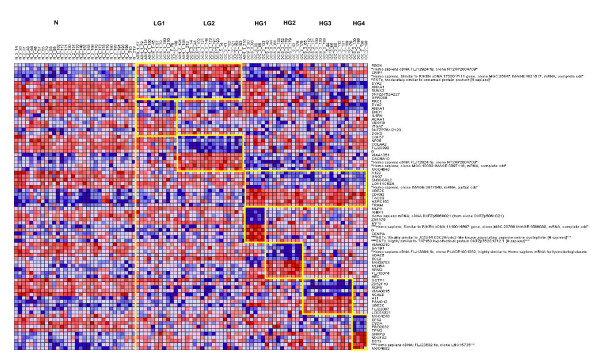
**Subtype heatmap using the top 10 markers**: Red/green represent up/down regulation relative to black. Each subgroup is shown in a framed box to identify its samples and distinguish gene markers. The signatures of the genes specific to each subtype stand out distinctly compared to all other subtypes.

Figure 4 presents a heatmap showing the classification into subtypes using top 10 gene markers for each. Each set of markers distinguished a given subtype from all the other subtypes with an accuracy above 90% in leave-one-out experiments for WV and kNN classification models (see Table 2). The signatures of the subgroups LG1-HG4 stand out clearly. Table 2 presents the sensitivity and specificity scores on leave-one-out cross-validation experiments for WV models. Note that the specificity ranges from 91–97%, and the sensitivity from 82–100%. The results for kNN are very similar. We note that these results may be slightly inflated because the subtypes were discovered on the same dataset on which we did the sensitivity/specificity analysis.

**Table 2:** Weighted voting classification accuracy : Sensitivity, specificity and accuracy of a weighted voting classifier in distinguishing samples in a given subtype from all other samples. The accuracy scores were computed using leave-one-out experiments. The genes used for classification were selected based on their collective power to accurately discriminate between a group and its complement.

Group	Sensitivity	Specificity	Accuracy
LG	89.29	90.77	90.32
LG1	81.82	91.46	90.32
LG2	100.00	90.79	92.47
HG	96.88	95.08	95.70
HG1	100.00	96.59	96.77
HG2	100.00	96.39	96.77
HG3	84.62	92.50	91.40
HG4	100.00	94.38	94.62

**Figure 5:**
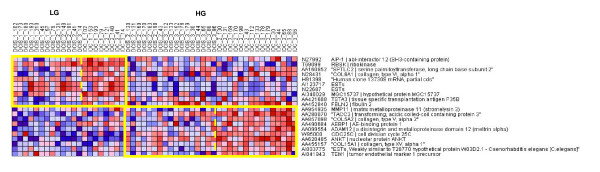
**Low-High grade progression heatmap**: Heatmap of expression levels of the top markers for progression from DCIS to IDC in the low grade and high grade tumor subgroups. In each subtype, we use the upregulated genes which have good FDR under WV to stratify the samples. We show the 10 top genes for DCIS to IDC progression in LG and HG tumors. Since the sample sizes were small, the p values were computed using permutation tests and the FDR values were computed from these p values. The FDR values under WV for these genes are 0.6 for LG and 0.2 for HG.

**Figure 6:**
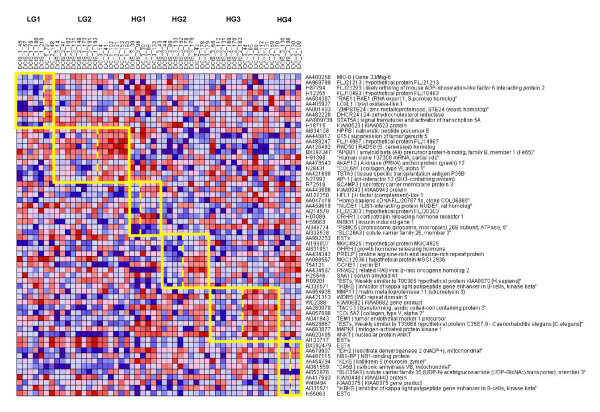
**DCIS to IDC progression heatmap**: Heatmap of expression levels of the top 10 upregulated genes for progression from DCIS to IDC for each subtype. Each subgroup is in a framed box to identify its samples and distinguish gene markers. Since the sample sizes are small, the p values were computed using permutation tests and the FDR rates inferred from these p values. The FDR rates under WV for these genes are: 0.02 for LG1, 0.2 for LG2, 0.2 for HG1, 0.5 for HG2, 0.06 for HG3 and 0.002 for HG4.

**Figure 7:**
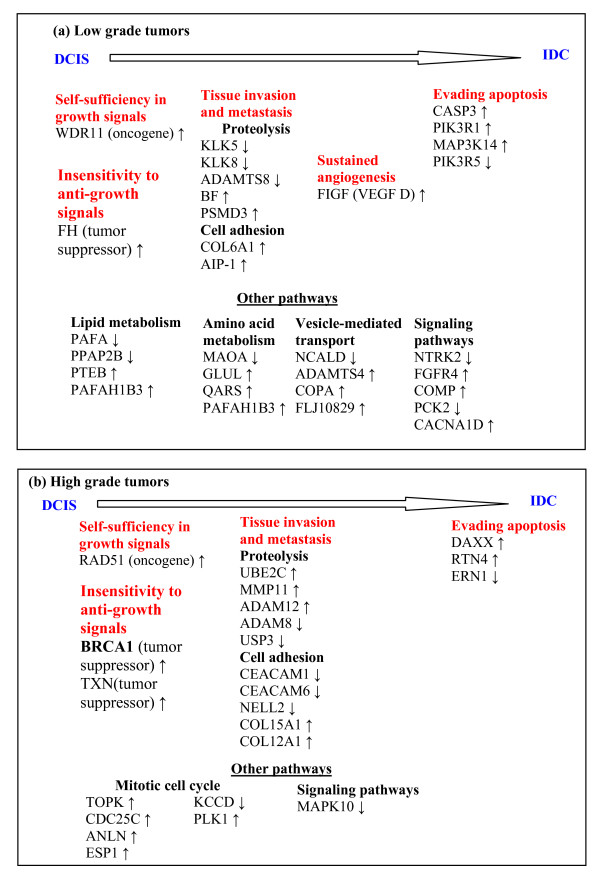
**Pathways affected in low and high-grade tumors**. Progression models for low and high grade tumors identified from functional analysis of genes characteristic of subtypes. Marker genes were placed into Hanahan-Weinberg [16] categories which are shown in red. Our results are in general agreement with the expectation that activation of oncogenes and loss of tumor suppressor genes are early events seen in low grade tumors and induction of angiogenesis is an early to mid-stage event seen in high grade tumors [23].

Figures 5 and 6 present heatmaps using the top 10 upregulated markers which classify the tumors by grade and stage respectively. Since the sample sizes are small, the p values for each classification were obtained using permutation experiments and the FDR rates inferred from these. The FDR values for the genes in Figure 5 are 0.6 for LG, 0.2 for HG and for the genes in Figure 6 are 0.02 for LG1, 0.2 for LG2, 0.2 for HG1, 0.5 for HG2, 0.06 for HG3 and 0.002 for HG4. It should be noted that the sample sizes in this study are small. Consequently, these results are to be considered as hypothesis generating. All these results should be validated on larger data sets. Figure 7 maps the genes identified for progression in different grades into pathways for disease progression using the classification of Hanahan and Weinberg [16,17].

## Discussion and conclusion

The use of ensemble consensus clustering is absolutely critical to distinguish the subtypes. PCA by itself could identify a collection of useful markers, but could not identify the rich stratification discovered by consensus ensemble *k*-clustering. Hierarchical clustering by itself would separate the samples into clusters but the stratification would be very sensitive to bootstrap, indicating that the clusters are unstable to data perturbation. Robustness of clustering is only obtained by averaging over many clustering techniques and data perturbations as is done in the ensemble consensus clustering technique used here.

Our results show that progression of the disease from non-invasive to invasive status occurs along different pathways. Progression in the low-grade groups seems to correlate with changes in metabolic and transportation pathways, while in the high grade groups it is related to alterations in cell-cycle and signaling pathways, with distinct subsets of genes involved in each.

Table 3 presents a summary of the significant pathways involved in the low-and high grade subgroups. We find that the differences between the levels in the DCIS and IDC groups are quite subtle and the accuracy of leave-one-out experiments of simple WV models trained to distinguish between DCIS and IDC in each group ranges between 60–70%.

**Table 3:** Pathways enriched in subtype clusters: Enriched functional pathways in low and high grade tumor groups and within subtypes using DAVID [36] using a cutoff p < 0.01.

**Group**	**Enriched pathway**
LG	lipid metabolism, transcriptional regulation, vesicle-mediated transport, amino-acid and derivative metabolism
LG1	small GTPase, mediated signal transduction, intracellular trafficking and vesicular transport
LG2	proteolysis collagens mRNA processing
HG	mitotic cell cycle, ATM signaling pathway, role of BRCA1, BRCA2 and ATR in cancer susceptibility, cell cycle: G2/M checkpoint
HG1	ion transport
HG2	cell cycle proteolysis
HG3	collagens proteolysis
HG4	proteolysis

The main observation of the original paper of Ma et al [10] was that the molecular signature of breast cancer is already present in the early (ADH) stage of the disease. The genes that distinguish ADH from Normal progressively change their levels away from Normal as the disease progresses to DCIS and IDC. They also noticed that that breast cancer progression is defined by distinct markers for low and high grade tumors. Our results, particularly the hierarchy we see when the data is grouped into *k *= 2,3,...7 clusters (Figure 2) agree with this observation.

Our methods identified six different subtypes of breast cancer with distinct patterns of progression. Looking at the histopathology of the samples in the clusters identified by our analysis, four of these subtypes (LG1, LG2, HG3, HG4) have a strongly Luminal signature (predominantly ER+, PR+, Her2-); one subtype (HG1) had the triple negative (ER-, PR-, Her2-) characteristic of the Basal-like subtype, and one subtype (HG2) had a predominantly Her2+ signature (mixed ER, mostly Her2+). The validation of these subtypes on a larger dataset with more genes is currently underway.

At *k *= 7, each of the six BCA clusters always contained samples in both DCIS and IDC stages *from the same patient*. This strong heterogeneity in the genetic signature of subtypes suggests that breast cancer is composed of distinct disease subtypes that develop early and progress along different pathways because progression within a subtype is less distinct than the subtypes themselves. Treatment decisions may benefit by taking account of these subtypes in addition to the current practice of using the markers ER, PR and Her2.

## Methods

Consensus ensemble clustering [18,19] was applied to the projection of the data on the genes identified by PCA to divide the data successively into *k *= 2, 3,..., *k*_opt _clusters which were made insensitive to data and clustering method perturbation using consensus ensemble clustering (see below). To maintain sensitivity to subtle genetic signals, we used the full set of genes on the samples after each *k *level clustering to find the best pool of genes that distinguished a cluster from other clusters. This non-stringent selection was motivated by the expectation that the key genes altered in disease pathways are likely to have subtle alterations in their expression levels and may not necessarily be the same genes that are best to distinguish the clusters. On this larger set of genes for each *k*, we identified two sub-classes. The first set distinguished each cluster from its complement. The second set defined progression from non-invasive to invasive disease. Finally, we used annotated databases to identify the functional pathways that are most representative of the clusters identified. Each of these steps is described in detail below.

### Data normalization and imputation

The genes were normalized by first applying a robust nonlinear local regression method as described in [10] and then by applying a global normalization procedure which consists of subtracting the median of each gene across the arrays. 13 genes had missing values in 13–15% of the samples and were discarded. 105 genes had missing entries for up to 5% of the samples. These missing entries were imputed using a dynamical *k*NN approach [20].

### Principal Component Analysis (PCA)

Principal Component Analysis or PCA [12,21,22] was used to retain those genes in the dataset that contribute most to its variance. PCA was applied to the expression matrix *E*_*ij *_whose the rows were the 93 samples and whose columns were the 1927 genes that survived after robust imputation of missing data. The analysis was done by a singular value decomposition of this matrix after it was centered and scaled to mean 0 and variance 1 per column. From the eigenvectors of the largest eigenvalues that accounted for 85% of the variation in the data we selected the subset of genes with coefficients in the top 25% in absolute value in these eigenvectors. This collection of genes was further used to find robust clusters in the data.

### Ensemble consensus *k*-clustering

Using the genes from PCA, we first identified the optimal number of clusters using gap statistics [11] and silhouette scores [12]. Next, we applied an ensemble consensus *k*-clustering approach (initiated by [18] and [19]) to group the samples into the optimum number of clusters. The ensemble consensus clustering integrates the results of various clustering techniques across sample data perturbations into a pairwise agreement matrix which is used to partition the samples into the optimum number of clusters.

The overall technique has two distinct parts: (1) a method which generates a collection of clustering solutions using different methods applied to many perturbations of the data, and (2) a consensus function that combines the clusters found to produce a single output clustering of the data. The approach used in our paper is summarized below.

**Step 1. **150 datasets were created from the imputed data restricted to the 207 significant genes identified by PCA. 50 datasets came from bootstrapping the samples, 50 from bootstrapping genes and 50 by first projecting the data on bootstrapped genes and then by further bootstrapping on samples.

**Step 2. **The optimal number of clusters *k*_opt_was inferred (*a priori*) using the gap statistic and silhouette scores.

**Step 3. ***k *= 2,..., *k*_opt _clusters were created using representative methods from the three major classes:

(i) *Partitioning*: partition around medoids (PAM) [12], *k*-means [23] and graph partitioning [24].

*(ii) Agglomerative*: hierarchical clustering based on average linkage, complete linkage and Ward metric [12] as well as bagglo, which is a hybrid agglomerative method developed by [24].

*(iii) Probabilistic*: expectation maximization (EM) method [25], entropy-based-clustering (ENCLUST) [26], clustering on subsets of attributes (COSA) [27].

**Step 4. **Each clustering method was applied 50 times with different parameter initialization on the full dataset, and once on each of the 150 datasets from Step 1. From the 200 resulting clusters, we constructed an agreement matrix of size *N*_sample _× *N*_sample _for each method, whose entries ^*m*^*ij *represented the fraction of times a pair of samples (*i, j*) occurred in the same cluster out of the number of times the pair was selected in the 200 datasets. Here *N*_sample _denotes the number of samples in the dataset.

**Step 5. **For each *k*, the agreement results of Step 4 were averaged across the clustering techniques. The samples were then sorted such that those with the highest pairwise agreement appeared along the diagonal of the agreement matrix in *k *blocks. We applied simulated annealing to find the *k *optimal clusters for which the average internal similarity (within each cluster) minus the average pairwise similarity (between all pairs of clusters) has a local maximum value.

### Identification of gene markers within clusters

We now used the full collection of genes on each of the clusters identified at each *k *by consensus ensemble clustering. The markers were chosen to discriminate between two classes: class 1 = the group of interest (ie, the entire cluster), class 0 = the samples not included in the group of interest (ie, the complement of the cluster). The best markers were identified in two steps.

**Step 1. **A large pool of genes which distinguished the two labeled classes was selected based on a variant of the t-test statistic called the signal to noise ratio (SNR) [28] with a permutation p-value of 0.1 and a False Discovery Rate (FDR) [29] of 0.5. The SNR statistic computes the difference of the means in each of two classes scaled by the sum of the standard deviations: SNR = (μ_0 _- μ_1_)/(σ_0 _+ σ_1_), where μ_0 _is the mean of class 0 and σ_0 _is the standard deviation of class 0 and so on. The t-test statistic is the same as the SNR except that the denominator is (σ_0_^2 ^+ σ_1_^2^)^1/2^. Since (σ_0 _+ σ_1_) > (σ_0_^2 ^+ σ_1_^2^)^1/2^) SNR penalizes features that have higher variance in each class more than those features that have a high variance in one class and a low variance in another. This bias is particularly useful in distinguishing genes which are altered in normal/disease or stage/grade progression. For example, in the normal/disease case, the pathway in which the gene is involved is working correctly in one class, and hence is regulated strictly (has low variance) while in the other class, the pathway is compromised and the gene is less well regulated (has high variation).

**Step 2. **From the larger pool of genes from Step 1, we identified the best genes correlated with the class label using stringent criteria which combined (a) a permutation p-value of 0.05 (b) stability to sample perturbation through bootstrapping (c) stability to leave-one-out experiments in top 25% genes selected by weighted voting and kNN classifiers which distinguish the two classes with specificity and sensitivity above 0.75. This analysis was done using the software GenePattern from the Broad Institute [30].

### Identification of pathways and biological/functional categories

We used the bioinformatics public resources DAVID [31], iHOP [32], and MatchMiner [33]. We also used 14 functional annotation sources including KEGG and GO annotations, Biocarta pathways, linked to DAVID as well as the Functional Classification Tool implemented in DAVID. The Functional Classification Tool groups genes based on functional similarity. It uses Kappa statistics [31] which is an index that compares the agreement against the possibility that it appeared by chance. Thus,

κ=Observed agreement - Chance agreement1 - Chance agreement.
 MathType@MTEF@5@5@+=feaafiart1ev1aaatCvAUfKttLearuWrP9MDH5MBPbIqV92AaeXatLxBI9gBaebbnrfifHhDYfgasaacH8akY=wiFfYdH8Gipec8Eeeu0xXdbba9frFj0=OqFfea0dXdd9vqai=hGuQ8kuc9pgc9s8qqaq=dirpe0xb9q8qiLsFr0=vr0=vr0dc8meaabaqaciaacaGaaeqabaqabeGadaaakeaacqaH6oWAcqGH9aqpdaWcaaqaaiabb+eapjabbkgaIjabbohaZjabbwgaLjabbkhaYjabbAha2jabbwgaLjabbsgaKjabbccaGiabbggaHjabbEgaNjabbkhaYjabbwgaLjabbwgaLjabb2gaTjabbwgaLjabb6gaUjabbsha0jabbccaGiabb2caTiabbccaGiabboeadjabbIgaOjabbggaHjabb6gaUjabbogaJjabbwgaLjabbccaGiabbggaHjabbEgaNjabbkhaYjabbwgaLjabbwgaLjabb2gaTjabbwgaLjabb6gaUjabbsha0bqaaiabbgdaXiabbccaGiabb2caTiabbccaGiabboeadjabbIgaOjabbggaHjabb6gaUjabbogaJjabbwgaLjabbccaGiabbggaHjabbEgaNjabbkhaYjabbwgaLjabbwgaLjabb2gaTjabbwgaLjabb6gaUjabbsha0baacqGGUaGlaaa@7709@

The Kappa statistic can be thought of as the chance-corrected proportional agreement, and possible values range from +1 (perfect agreement) to 0 (no agreement above chance) to -1 (complete disagreement). The algorithm first generates a gene-to-gene similarity matrix (genes in rows and functional terms in columns) based on shared functional annotation. The matrix is made from binary entries. If a gene is annotated in a term, the term entry is 1, if not then the entry is 0. The algorithm adopts the kappa statistic to quantitatively measure the degree to which genes share similar annotation terms. The higher the value of κ, the stronger the agreement. The Fuzzy Heuristic Partition algorithm [31], which allows a gene to participate in more than one cluster, was used to classify highly related genes into functionally related groups.

### Additional Validation

An important question is whether the gene lists found for the subtypes identified in [5-7] and elsewhere as well as in the present paper are sufficiently stable to have clinical significance (be useful to analyze metastatic risk and have consequences for drug discovery). In a recent paper [32], a subset of the present authors reanalyzed the data of [5] using the robust clustering techniques described above. It was found that whereas there was sufficient clustering to verify that the subtypes Luminal A, Luminal B, Basal-like and Her2+ formed distinct clusters, the choice of genes in [5] was too limited to allow a detailed study of pathways specific to each subtype. The number of gene expressions available in the dataset used in the present paper is also rather limited (~1200) to make any detailed analysis of pathways. Consequently, we have analyzed a bigger dataset from a recent study [34] consisting of microarray data on U133a Affymetrix chips (~22,000 genes) for 286 node negative patients treated with radiation and surgery with 10 year clinical follow-up. This analysis will be reported in a separate publication [35] and completely confirms the subtypes and gene signatures found in the present paper.

## Authors' contributions

GSD and CD acquired the data; GSD, SG and CD provided the biological interpretation of the results. GSD and DS provided the annotations for the gene markers identified in the study. GA and GB designed the computational approach. GA performed the computational analysis. GB wrote the manuscript. GB, SG and CD supervised the research group. PT and JM worked on the GSEA and consensus clustering analysis. We thank Dr. Stefano Monti for discussions about estimating the number of clusters.

## Supplementary Material

Additional file 1 **Supplementary Table 1**. A listing of the samples used in our study. Clinical stage and grade are as provided in the data of Ma et al (PNAS 2003). The column "Subtype" presents our classification into Normals and six disease subtypes (Basal, HER2+, Luminal A, Luminal B1, Luminal B2 and Luminal B3) based on PCA and Clustering. The remaining columns list clinical information as provided in the data from Ma et al (PNAS 2003). Click here for file
