# Supplementary material for: Portraits of breast cancer progression
Source: BMC Bioinformatics. 2007 Aug 6;8:291. doi: 10.1186/1471-2105-8-291 (PMC1978212; doi:10.1186/1471-2105-8-291)
Supplement: Additional file 1 — Supplementary Table 1. A listing of the samples used in our study. Clinical stage and grade are as provided in the data of Ma et al (PNAS 2003). The column "Subtype" presents our classification into Normals and six disease subtypes (Basal, HER2+, Luminal A, Luminal B1, Luminal B2 and Luminal B3) based on PCA and Clustering. The remaining columns list clinical information as provided in the data from Ma et al (PNAS 2003). [file 1471-2105-8-291-S1.pdf]

Supplementary Table 1. A listing of the samples used in our study. Clinical stage and grade are as provided in the data of Ma et al (PNAS 2003). The column "Subtype" presents our classification into Normals and six disease subtypes (Basal, HER2+, Luminal A, Luminal B1, Luminal B2 and Luminal B3) based on PCA and Clustering. The remaining columns list clinical information as provided in the data by Ma et al (PNAS 2003).

#	Case id	Sample label	Stage	Grade	Subtype	**Age**	**ER**	**PR**	**HER2**	**Node***
62	30	DCIS_3_30	DCIS	3	Basal	47	Neg	Neg	Neg	Pos
63	96	DCIS_3_96	DCIS	3	Basal	31	Neg	Neg	Neg	Pos
64	30	IDC_3_30	IDC	3	Basal	47	Neg	Neg	Neg	Pos
65	96	IDC_3_96	IDC	3	Basal	31	Neg	Neg	Neg	Pos
66	133	IDC_3_133	IDC	3	Basal	44	Neg	Neg	Pos	Pos
67	43	DCIS_2_43	DCIS	2	HER2+	53	Pos	Neg	Neg	Pos
68	44	DCIS_3_44	DCIS	3	HER2+	28	Pos	Pos	Neg	Neg
69	88	DCIS_3_88	DCIS	3	HER2+	35	Pos	Pos	ND	Pos
70	133	DCIS_3_133	DCIS	3	HER2+	44	Neg	Neg	Pos	Pos
71	152	DCIS_3_152	DCIS	3	HER2+	NA	NA	NA	NA	NA
72	178	DCIS_3_178	DCIS	3	HER2+	43	Pos	Pos	Pos	Pos
73	179	DCIS_3_179	DCIS	3	HER2+	37	Neg	Neg	Pos-FISH	Pos
74	43	IDC_2_43	IDC	2	HER2+	53	Pos	Neg	Neg	Pos
75	178	IDC_3_178	IDC	3	HER2+	43	Pos	Pos	Pos	Pos
76	179	IDC_3_179	IDC	3	HER2+	37	Neg	Neg	Pos-FISH	Pos
77	65	DCIS_3_65	DCIS	3	Luminal B2	39	Pos	Pos	Neg	Neg
78	112	DCIS_3_112	DCIS	3	Luminal B2	31	Neg	Pos	Neg	Pos
79	131	DCIS_2_131	DCIS	2	Luminal B2	37	Pos	Pos	Pos	Pos
80	169	DCIS_2_169	DCIS	2	Luminal B2	34	Pos	Pos	Neg	Pos
81	170	DCIS_2_170	DCIS	2	Luminal B2	44	Pos	Pos	Pos-FISH	Pos
82	183	DCIS_2_183	DCIS	2	Luminal B2	46	ND	ND	ND	Pos
83	44	IDC_3_44	IDC	3	Luminal B2	28	Pos	Pos	Neg	Neg
84	65	IDC_3_65	IDC	3	Luminal B2	39	Pos	Pos	Neg	Neg
85	88	IDC_3_88	IDC	3	Luminal B2	35	Pos	Pos	ND	Pos
86	112	IDC_3_112	IDC	3	Luminal B2	31	Neg	Pos	Neg	Pos
87	131	IDC_2_131	IDC	2	Luminal B2	37	Pos	Pos	Pos	Pos
88	169	IDC_2_169	IDC	2	Luminal B2	34	Pos	Pos	Neg	Pos
89	170	IDC_2_170	IDC	2	Luminal B2	44	Pos	Pos	Pos-FISH	Pos
90	130	DCIS_2_130	DCIS	2	Luminal B3	54	Pos	Pos	Neg	Pos
91	198	DCIS_2_198	DCIS	2	Luminal B3	30	Pos	Pos	Neg	Neg
92	130	IDC_2_130	IDC	2	Luminal B3	54	Pos	Pos	Neg	Pos
93	198	IDC_2_198	IDC	2	Luminal B3	30	Pos	Pos	Neg	Neg
33	180	ADH_1_180	ADH	1	Luminal A	46	Pos	Pos	Neg	Pos
34	57	ADH_1_57	ADH	1	Luminal A	NA	NA	NA	NA	NA
35	79	ADH_1_79	ADH	1	Luminal A	54	Pos	Pos	Neg	Pos
36	131	ADH_2_131	ADH	2	Luminal A	37	Pos	Pos	Pos	Pos
41	45	DCIS_1_45	DCIS	1	Luminal A	36	Pos	Neg	Neg	Neg
42	57	DCIS_1_57	DCIS	1	Luminal A	NA	NA	NA	NA	NA
43	79	DCIS_1_79	DCIS	1	Luminal A	54	Pos	Pos	Neg	Pos
44	180	DCIS_1_180	DCIS	1	Luminal A	46	Pos	Pos	Neg	Pos
45	210	DCIS_1_210	DCIS	1	Luminal A	NA	NA	NA	NA	NA

46	79	IDC_1_79	IDC	1	Luminal A	54	Pos	Pos	Neg	Pos
47	148	IDC_2_148	IDC	2	Luminal A	42	Pos	Pos	Neg	Pos
37	213	ADH__213	ADH	NA	Luminal B1	NA	NA	NA	NA	NA
38	193	ADH_1_193	ADH	1	Luminal B1	45	Pos	Pos	Neg	Pos
39	191	ADH_2_191	ADH	2	Luminal B1	NA	NA	NA	NA	NA
48	14	DCIS_3_14	DCIS	1	Luminal B1	44	Pos	Pos	ND	Pos
49	41	DCIS_2_41	DCIS	2	Luminal B1	55	Pos	Pos	ND	Neg
50	102	DCIS_1_102	DCIS	1	Luminal B1	55	Pos	Neg	Neg	Pos
51	121	DCIS_2_121	DCIS	2	Luminal B1	45	Pos	Pos	Pos	Pos
52	148	DCIS_2_148	DCIS	2	Luminal B1	42	Pos	Pos	Neg	Pos
53	173	DCIS_1_173	DCIS	1	Luminal B1	52	Pos	Pos	Neg	Neg
54	191	DCIS_2_191	DCIS	2	Luminal B1	NA	NA	NA	NA	NA
55	193	DCIS_1_193	DCIS	1	Luminal B1	45	Pos	Pos	Neg	Pos
56	14	IDC_3_14	IDC	1	Luminal B1	44	Pos	Pos	ND	Pos
57	41	IDC_2_41	IDC	2	Luminal B1	55	Pos	Pos	ND	Neg
58	102	IDC_1_102	IDC	1	Luminal B1	55	Pos	Neg	Neg	Pos
59	121	IDC_2_121	IDC	2	Luminal B1	45	Pos	Pos	Pos	Pos
60	153	IDC_1_153	IDC	1	Luminal B1	46	Pos	Pos	Pos	Pos
61	193	IDC_1_193	IDC	1	Luminal B1	45	Pos	Pos	Neg	Pos
1	210	N__210	N	1	Normal	NA	NA	NA	NA	NA
2	213	N__213	N	NA	Normal	NA	NA	NA	NA	NA
3	102	N_1_102	N	1	Normal	55	Pos	Neg	Neg	Pos
4	153	N_1_153	N	1	Normal	46	Pos	Pos	Pos	Pos
5	173	N_1_173	N	1	Normal	52	Pos	Pos	Neg	Neg
6	180	N_1_180	N	1	Normal	46	Pos	Pos	Neg	Pos
7	193	N_1_193	N	1	Normal	45	Pos	Pos	Neg	Pos
8	45	N_1_45	N	1	Normal	36	Pos	Neg	Neg	Neg
9	57	N_1_57	N	1	Normal	NA	NA	NA	NA	NA
10	79	N_1_79	N	1	Normal	54	Pos	Pos	Neg	Pos
11	121	N_2_121	N	2	Normal	45	Pos	Pos	Pos	Pos
12	130	N_2_130	N	2	Normal	54	Pos	Pos	Neg	Pos
13	131	N_2_131	N	2	Normal	37	Pos	Pos	Pos	Pos
14	148	N_2_148	N	2	Normal	42	Pos	Pos	Neg	Pos
15	169	N_2_169	N	2	Normal	34	Pos	Pos	Neg	Pos
16	170	N_2_170	N	2	Normal	44	Pos	Pos	Pos-FISH	Pos
17	183	N_2_183	N	2	Normal	46	ND	ND	ND	Pos
18	191	N_2_191	N	2	Normal	NA	NA	NA	NA	NA
19	198	N_2_198	N	2	Normal	30	Pos	Pos	Neg	Neg
20	41	N_2_41	N	2	Normal	55	Pos	Pos	ND	Neg
21	43	N_2_43	N	2	Normal	53	Pos	Neg	Neg	Pos
22	112	N_3_112	N	3	Normal	31	Neg	Pos	Neg	Pos
23	133	N_3_133	N	3	Normal	44	Neg	Neg	Pos	Pos
24	14	N_3_14	N	1	Normal	44	Pos	Pos	ND	Pos
25	152	N_3_152	N	3	Normal	NA	NA	NA	NA	NA
26	178	N_3_178	N	3	Normal	43	Pos	Pos	Pos	Pos
27	179	N_3_179	N	3	Normal	37	Neg	Neg	Pos-FISH	Pos
28	30	N_3_30	N	3	Normal	47	Neg	Neg	Neg	Pos
29	44	N_3_44	N	3	Normal	28	Pos	Pos	Neg	Neg
30	65	N_3_65	N	3	Normal	39	Pos	Pos	Neg	Neg
31	88	N_3_88	N	3	Normal	35	Pos	Pos	ND	Pos
32	96	N_3_96	N	3	Normal	31	Neg	Neg	Neg	Pos
40	210	ADH_1_210	ADH	1	Normal	NA	NA	NA	NA	NA
